# Photodynamic Diagnosis and Therapy in Non-Muscle-Invasive Bladder Cancer

**DOI:** 10.3390/cancers16132299

**Published:** 2024-06-22

**Authors:** Atsushi Kurabayashi, Hideo Fukuhara, Kaoru Furihata, Waka Iwashita, Mutsuo Furihata, Keiji Inoue

**Affiliations:** 1 Department of Pathology, Kochi Medical School, Nankoku 783-8505, Kochi, Japan; kfurihata@kochi-u.ac.jp (K.F.); w.iwashita@kochi-u.ac.jp (W.I.); furiham@kochi-u.ac.jp (M.F.); 2 Department of Urology, Kochi Medical School, Nankoku 783-8505, Kochi, Japan; jm-fukuhara@kochi-u.ac.jp (H.F.); keiji@kochi-u.ac.jp (K.I.)

**Keywords:** non-muscle-invasive bladder cancer, transurethral resection of bladder tumor, 5-Aminolevulinic acid, photodynamic diagnosis, photodynamic therapy

## Abstract

**Simple Summary:**

Bladder cancer (BC) possesses distinct molecular profiles that influence progression depending on its biological nature and delivered treatment intensity. Muscle-invasive BC (MIBC) and non-MIBC (NMIBC) demonstrate great intrinsic heterogeneity regarding different prognoses, survival, progression, and treatment outcomes. Transurethral resection of bladder tumor (TURBT) is the standard treatment for NMIBC. The high risks of disease recurrence from residual tumor and progression after TURBT in NMIBC are well known. A new-generation photosensitizer, 5-Aminolevulinic acid (5-ALA), with a high tumor specificity, has been studied for detecting precise tumor areas. Moreover, it has been applied for treatment by producing its cytotoxic reactive oxygen species, as well as screening for urological carcinomas by excreting porphyrin in the blood and urine. Thus, 5-ALA may contribute to the inclusive treatment of NMIBC.

**Abstract:**

Bladder cancer (BC) possesses distinct molecular profiles that influence progression depending on its biological nature and delivered treatment intensity. Muscle-invasive BC (MIBC) and non-MIBC (NMIBC) demonstrate great intrinsic heterogeneity regarding different prognoses, survival, progression, and treatment outcomes. Transurethral resection of bladder tumor (TURBT) is the standard of care in treating NMIBC and serves both diagnostic and therapeutic purposes despite the prevalent recurrence and progression among many patients. In particular, flat urothelial carcinoma in situ and urothelial carcinoma with lamina propria invasion are the major precursors of MIBC. A new-generation photosensitizer, 5-Aminolevulinic acid (5-ALA), demonstrates high tumor specificity by illuminating the tumor lesion with a specific wavelength of light to produce fluorescence and has been studied for photodynamic diagnosis to detect precise tumor areas by TURBT. Additionally, it has been applied for treatment by producing its cytotoxic reactive oxygen species, as well as screening for urological carcinomas by excreting porphyrin in the blood and urine. Moreover, 5-ALA may contribute to screening before and after TURBT in NMIBC. Here, we summarize the updated evidence and ongoing research on photodynamic technology for NMIBC, providing insight into the potential for improving patient outcomes.

## 1. Introduction

Bladder cancer (BC) is one of the prevalent tumors that cause health issues globally [1,2]. Cigarette smoking is the most prominent risk factor for developing BC in most countries [3,4]. Overall, BC, with urothelial carcinoma (UC) as the prevalent histology, comprises two distinct molecular subtypes based on each molecular heterogeneity and clinical staging: muscle-invasive BC (MIBC), including stages T2 (muscularis propria invasion), T3 (perivesical fat invasion), and T4 (adjacent organ involvement), and non-MIBS (NMIBC), including stages Tis (flat urothelial carcinoma in situ [CIS]), Ta (NMIBC occurs as a papillary lesion), and T1 (NMIBC with lamina propria invasion), consisting of 80% BC. The NMIBC region of clonally related bladder tumors is frequently treatable [5]. The 5-year survival rate for NMIBC demonstrated no marked changes despite advancements in research and care, and the frequent NMIBC recurrence has burdened public health systems [6,7,8].

Understanding the molecular principles of NMIBC tumorigenesis is crucial for the development of effective preventive measures. Sequencing and gene expression studies have guided the discovery of numerous genetic alterations and revealed several distinct molecular signatures and subtypes, thereby providing a more accurate prediction of disease progression in BC. De novo CIS, as the primary CIS, constitutes less than 3% of all urothelial neoplasms. It is reported that most concurrent CIS are accompanied by invasive UC (frequency: 45–65%) and papillary UC (frequency: 7–15%) [9]. Furthermore, CIS may be detected in 90% of urothelial neoplasms during the observation period (secondary CIS) [10,11]. CIS patients with prior or concomitant papillary bladder neoplasia are more likely to present with a poor prognosis or die of the disease compared to those with primary CIS [11].

Transurethral resection of bladder tumor (TURBT) demonstrates both diagnostic and therapeutic functions in effective NMIBC management. TURBT, followed by intravesical Bacillus Calmette–Guérin (BCG) administration, is the standard of care in flat-lesion CIS despite prevalent recurrence and progression among many patients [9,12]. Intravesical BCG treatment seems to be preferred for de novo CIS [13]. In a previous study, 95% of superficial bladder tumors that did not respond to BCG progressed in 5 years, compared to 19% in the responsive group [14]. The use of photodynamic technology for the future management of challenging cases enables the safe and effective performance of TURBT. CIS is usually difficult to detect and differentiate from inflammatory lesions under a cystoscopic examination [15]. Advanced cystoscopy technologies, such as narrow-band imaging, photodynamic diagnosis, and image 1S, are required to improve BC detection. Novel techniques have increased the accuracy of transurethral surgery.

This review aims to summarize current evidence and recent advances in the molecular and translational aspects of BC biology and discuss potential or future clinical applications in the management of NMIBC.

## 2. Common Genetic Alterations in NMIBC

Deletions of chromosome 9, frequently found in NMIBC, include the CDKN2A locus (9p21) that encodes p16 and p14ARF, which are RB (retinoblastoma gene) and p53 pathway regulators, respectively. The loss of TSC1 (9q34), which is an mTOR signaling regulator associated with the upregulated expression of mTOR targets [16], such as telomerase reverse transcriptase (TERT) activity, has been observed in NMIBC. Overexpressed TERT upregulates oncogenic signaling pathways [17], which are crucial in maintaining tumor immortality and contributing to tumor progression in BC, to maintain telomere integrity [18,19,20,21]. The promoter mutations of TERT are chief genetic alterations occurring with a frequency of 70–80% in patients with BC [22,23,24,25]. Other copy number changes in NMIBC (8–22%) include increases in 1q, 5p, 18q, 20p, and 20q and decreases in 8p, 11p, 17p, and 18q, especially in stage T1 tumors [26], which are more commonly detected in NMIBC [27].

Point mutations in FGFR3 (S249C), predicted to result in apolipoprotein B mRNA-editing catalytic polypeptide-like (APOBEC) activity [28] as an initial event, are the most common genetic alterations associated with low tumor grades and stages in NMIBC [16,29,30,31,32]. APOBEC targets PIK3CA mutations that are present in <30% of NMIBC [33]. RAS or FGFR3 mutation, found in Ta tumors, are mutually exclusive and associated with low tumor grades and stages [16,33]. These data indicate that both PI3K and RAS–MAPK signaling pathways are usually activated in NMIBC. *ERBB2* and *ERBB3* mutations that provide PI3K activation [34] are detected in <15% of T1 tumors. Micropapillary and plasmacytoid UC are biologically aggressive subtypes [5]. Micropapillary UC has a disproportionately higher rate of *ERBB2* amplification than conventional UC [35,36,37], and plasmacytoid UC is defined by distinct *CDH1* mutations [38]. Inactivating mutations of *STAG 2* and the loss of its protein expression are observed in <30% of low-grade Ta tumors and fewer T1 tumors, often with *FGFR3*, *PIK3CA*, and/or *KDM6A* (chromatin regulator) mutations [33,39,40]. The exact roles of *FGFR3* mutations in NMIBC tumorigenesis remain unknown. However, mutant *FGFR3* induces the overgrowth of the cultured normal human urothelial cells at a confluence [41], indicating a potential contribution to urothelial hyperplasia in vivo. Foth et al. used a murine model to show that S249C mutation in *FGFR3* genes could initiate tumor progression by suppressing the acute inflammatory response as an antitumor response; additionally, the immune cells were perturbed in the tumor [42]. Based on this suppression of immune cells by *FGFR3* mutation, the authors speculated on the usefulness of combining FGFR3 inhibitors and BCG therapy as immune activation for tumors [42]. Critelli et al. reported that stronger associations with recurrences (odds ratio [OR], 2.51; 95% confidence interval [CI], 1.26–5.00; *p* = 0.01) and the number of recurrences (OR, 4.51; 95% CI, 1.27–16.06; *p* = 0.02) were observed in NMIBC, especially when mutations in *FGFR3* and *TERT* were combined [43]. 

Moreover, BC demonstrates significant epigenetic dysregulation, such as changes in DNA methylation [44]. Rink et al. reviewed the literature on the utility of circulating biomarkers, such as circulating tumor DNA (ctDNA) in plasma and urine samples in NMIBC patients, and suggested the use of biomarkers (e.g., the detection of *FGFR3* and *PIK3CA* mutations in plasma and urine and the methylation levels of EOMES, HOXA9, POU4F2, TWIST1, VIM, and ZNF154 in urine specimens) for disease surveillance in the future [45]. There is limited evidence on biomarkers in NMIBC. However, if frequent mutations and epigenetic modifications can be identified in small lesions, the references may benefit novel therapeutic approaches. Moreover, they could be used during urine and blood surveillance for both early detection and disease monitoring after treatment. 

## 3. Tumorigenesis Associated with NMIBC

Relative to treating NMIBC, Ta tumors, above all, have demonstrated good prognoses compared to T1 and Tis tumors, and their treatment plan is markedly different [1]. The use of tissue to predict progression from T1 to MIBC has been a subject of interest for some time. The American Joint Committee on Cancer (AJCC) indicated that T1 tumors showed that tumors in the lamina propria were related to a higher rate of progress based on many studies [46]. Moreover, a total tumor diameter cutoff of ≥2.3 mm was reported to be the best predictor of muscle-invasive disease progression. Additional non-histological considerations may be required for a lower false-positive threshold [47]. Additional molecular biological approaches (e.g., molecular and/or protein biomarkers) to predicting MIBC progression have been assessed in numerous studies [48,49]. These studies revealed that improved sequencing techniques have contributed to the understanding of BC as a heterogeneous disease and enabled the development of several biomarkers with the potential to help predict the treatment response and appropriate patient selection.

T1 tumors frequently share molecular characteristics with MIBC and are usually very different from low-grade Ta tumors [2,50,51]. High-grade BCs are either NMIBC or MIBC, of which T1 tumors prevalently progress to MIBC, requiring more aggressive clinical treatment with a close follow-up. In addition, both histopathological and molecular data indicate the CIS as the major MIBC precursor [52]. Although most Ta tumors originate from an urothelium that appears normal, some high-grade Ta tumors progress to T1 BC, which leads to MIBC. Alternatively, it is well known that low-grade Ta tumors recur in approximately 50% of cases [5], resulting in repeated recurrences following treatment.

**Figure 1:**
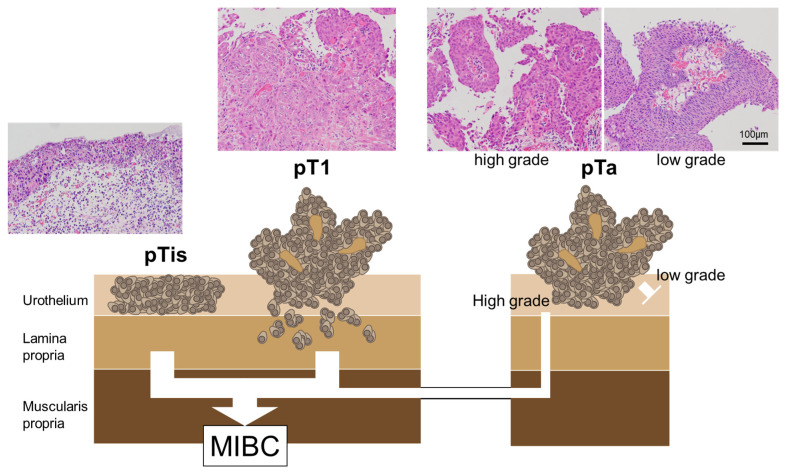
The correlation between pathological findings and MIBC progression in NMIBC. The CIS and pT1 BCs are the major MIBC precursors. pTis: flat urothelial CIS; pT1a: noninvasive papillary lesion; pT1: BCs with invasion of the lamina propria.

Regarding the mutational profiles of NMIBC, T1 tumors are more closely related to MIBC than Ta tumors; nonetheless, the mutational profiles of T1 tumors often contain both Ta-like and MIBC-like features [52]. There is no absolute pathogenic pathway for the progression from NMIBC to MIBC; these tumor categories involve nonoverlapping pathways [53,54]. Figure 1 summarizes the correlation between the pathological findings of NMIBC and the progress to MIBC.

For high-grade NMIBC that can progress to MIBC, it is important to accurately diagnose and treat the lesions at the Ta or Tis (CIS) stages. Furthermore, even in the case of low-grade Ta tumors, it is important to accurately diagnose the extent of the disease (e.g., micro-lesions) and perform complete resection. This requires accurate diagnostic and lesion visualization techniques. 

## 4. Pathological Diagnosis and Screening of NMIBC

### 4.1. Urine-Based Diagnosis

A prospective observational study revealed that approximately 75% of patients with BC present with non-symptomatic macrohematuria, with an increasing incidence alongside aging [55,56]. Patients with hematuria should be diagnosed based on the results of a urine-based examination (e.g., cytology). Urine cytology is a simple, minimally invasive, and inexpensive test with a high specificity for diagnosing BC [57]. Urine cytology needs to be evaluated using a small number of cell samples. The quality of urine cytology depends on the collection method and the number of cells collected. Therefore, appropriate evaluation standards for urine cytology are needed. As a result, the Paris System for Reporting Urinary Cytology was published in 2016 as a standardized system for reporting urine cytology [58]. Although the sensitivity of this analysis is not the highest, its specificity is high, especially in high-grade BC. Therefore, urine cytology remains a well-established technique in BC diagnosis compared to marker-based studies such as protein- or molecule-based urinalysis. [58,59,60,61,62,63,64]. If techniques that increase the sensitivity of tumor cell detection can be added to urine cytology, it will become a simple and even more powerful diagnostic tool for BC.

### 4.2. Blood-Based Diagnosis

The development of circulating cell-free DNA detection technology is a major advance in the field of innovative liquid biopsy diagnostics. Cell-free DNA with tumor-specific alterations is derived from dying (i.e., apoptotic) cells and released into the blood flow (ctDNA) [65]. The half-life of ct-DNA in cancer patients (e.g., colorectal cancer) is believed to be up to 2 h [66]. ctDNA tracking is useful for a real-time understanding of the tumor burden postoperatively and during chemotherapy. ctDNA sensitively and specifically identifies patients at an early risk, predicts treatment efficacy, and detects tumor metastasis earlier [67]. Thus, it is predicted that detecting micro-BC at a very early stage will be required, and the development of technology for visualizing micro-lesions is critical.

### 4.3. Cystoscopic Diagnosis

Cystoscopy is the absolute standard for diagnosing BC; however, conventional cystoscopy using white light (WL) may miss small and/or flat lesions, such as CIS. When a lesion is found by cystoscopy, TURBT is subsequently performed under anesthesia. Then, histopathological examination, including histopathological staging, is performed; however, the latter does not always correspond with clinical staging [68]. Therefore, to understand the patient’s condition, the secure detection of lesions by cystoscopy, appropriate sample collection, and accurate histopathological examination (e.g., urine cytology and an analysis of the biopsy or TURBT samples from visible lesions) are important.

### 4.4. Tissue-Based Diagnosis

As mentioned above, the pathological analysis of tissue samples by bladder biopsy or TURBT during cystoscopy is the most popular and useful procedure for diagnosing the presence, histology, and stage of BC. BCs are categorized into low and high grades [5]. The most common type is UC (>90%). The subtypes, such as pure squamous cell carcinoma (5%) and pure adenocarcinoma (0.5–2%), are far rarer compared to UC [5,69]. In addition, UC exhibits remarkable diversity and differentiates into various subtypes. In addition to conventional UC, the World Health Organization’s classification of tumors of the urinary system and male genital tract defines 13 subcategories of UC (e.g., squamous, glandular, or trophoblast differentiation; micropapillary UC; plasmacytoid UC; lymphoepithelioma-like UC). Each has molecular pathological features that are associated with immunohistochemical and therapeutic differences [5,35,36,37,38,70,71]. The UROMOL study categorized NMIBC into three classes (i.e., class 1: luminal-like signature; class 2: luminal-like, epithelial-mesenchymal transition [EMT], and cancer stem cell signatures; class 3: basal-like signature) [72]. High-grade, high-stage tumors (T2-4), the coexistence of CIS, and progression to MIBC are more common in class 2 and class 3 tumors than in class 1 tumors. Therefore, class 2 tumors have a poor prognosis compared with classes 1 and 3 [72]. Pathological diagnosis identifies the tumor staging based on the AJCC, currently in its eighth edition [46]. NMIBC occurs as papillary lesions (pTa) or flat urothelial CIS (pTis). Cancer patients with similar prognoses are grouped by using prognostic stage tables. Classification by invasion depth (i.e., pT1: lamina propria invasion; pT2: muscularis propria invasion; pT3: invasion to perivesical fat; pT4: invasion to adjacent organ) is associated with decreased survival [46]. As mentioned above, the histological morphology of a tumor indicates its future trend. Thus, detailed histological observation, including the coexistence of CIS complications, is crucial.

## 5. Management of NMIBC

### TURBT and en Bloc Resection

Histopathologic diagnosis and staging determination using TURBT samples are often difficult because of tissue fragmentation, degeneration from cauterization and crushing, and the loss of an objective orientation for determining the presence or absence of muscularis propria in the tissue. En bloc resection of bladder tumor (ERBT) is a technique for resecting bladder tumors, which improves the above-mentioned shortcomings of TURBT by resecting the tumor segment in one piece, facilitates sampling of the muscularis propria near the tumor base, and results in more informative pathological specimens. Dyrskjøt et al. reviewed the results from three randomized trials comparing ERBT and TURBT in ≤3 cm BT [52]. First, Gellioli et al. reported that ERBT was not inferior to TURBT in terms of the rate of muscularis propria present (94% vs. 95%, *n* = 248), but T1 substaging was feasible in 80% of the TURBT cases compared with 100% of the ERBT cases (*p* = 0.02) [73]. In the second trial, D’Andrea et al. reported that ERBT was superior to TURBT in the retrieval of muscularis propria (80.7% vs. 71.1%; *p* = 0.01; *n* = 452) [74]. Finally, Teoh et al. reported ERBT reduced the 1-year recurrence rates and progression rate compared to TURBT (28.5 % vs. 38.1%; *p* = 0.007 and 0% vs. 2.6%; 0.065, respectively; *n* = 276) [75].

Furthermore, intravesical chemotherapy (CT), along with the transurethral resection of the bladder, is quite effective in reducing disease recurrence [76,77].

Either way, increasing the detection rate of tumors may contribute to a better prognosis in BC by these procedures.

## 6. Photodynamic Diagnosis, Photodynamic Therapy, and Photodynamic Screening with 5-Aminolaevulinic Acid

CIS is difficult to detect and differentiate from inflammation. The prognosis of the patients is improved if the expanse of the lesion in the BC, especially in T1 tumors, and the flat lesion (CIS), which is the major precursor of MIBC, can be detected and resected exactly. Photodynamic diagnosis (PDD) is a technique that improves the detection rate of occult BC, such as small and/or flat lesions, during cystoscopy. Photodynamic therapy (PDT) for BC involves administering a photosensitizing agent via systemic injection or bladder instillation. This is followed by exposure to visible light of an appropriate wavelength to induce a photochemical reaction that generates tissue-damaging reactive and singlet oxygen species in situ. The amino acid, 5-aminolaevulinic acid (5-ALA), is a new-generation photosensitizer with a high tumor specificity [78].

Generally, 5-ALA is generated in plants and animals from glycine and succinyl CoA [78]. Fluorescent endogenous porphyrins, such as protoporphyrin IX (PpIX), are produced in mitochondria by 5-ALA, which is transported via ATP-binding cassette (ABC) subfamily B member 6. Ferrochelatase catalyzes the insertion of ferrous iron into PpIX to form heme and bilirubin. Ferrochelatase contributes to PpIX catabolism in addition to heme production. However, ferrochelatase is inactive in various cancer types, including BC. Thus, PpIX accumulates more in tumor cells than in healthy cells due to decreased ferrochelatase activity [78,79,80]. Activating the 5-ALA synthetic enzyme and 5-ALA influx transporter (peptide transporter 1) promotes PpIX production in tumor cells [71]. Additionally, ABC superfamily G member 2, which excretes PpIX, is inactivated in tumor cells, thereby downregulating PpIX excretion from cells [78,81,82].

PpIX is photoactive; it is excited by light irradiation at specific wavelengths (mainly visible blue light [375–445 nm]) and emits red fluorescence at 600–700 nm. Therefore, cancer cells can be accurately identified by administering ALA and detecting the PpIX fluorescence. PpIX is excited at lower excitation wavelengths in the red (600–740 nm) and green (450–580 nm) visible range. PpIX returns from the excited state to the ground state after absorbing the light, while releasing energy, which produces cytotoxic reactive oxygen species (ROS) in tumor cells. ROS damages mitochondria and induces the apoptosis of the tumor cells, causing cell death. This mechanism emphasizes the effects of PDT with ALA [78].

**Figure 2:**
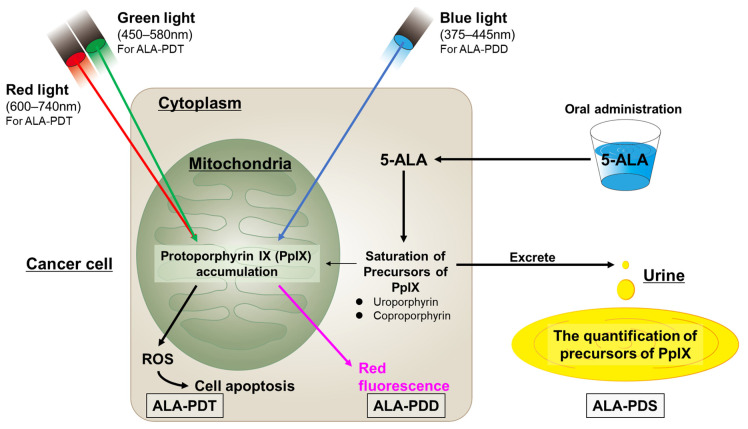
The mechanisms of ALA-PDD, ALA-PDT, and ALA-PDS.

PpIX accumulates in the mitochondria in the cancer cells. The excess PpIX causes the saturation of the porphyrin precursors of PpIX (i.e., uroporphyrins and coproporphyrins) and promotes their excretion into the blood and urine, thus raising their amounts in cancer patients. Photodynamic screening (PDS) is performed to measure the levels of these precursors [83]. Figure 2 summarizes the mechanisms of ALA-PDD, -PDT, and -PDS.

The 5-ALA is administered via oral and transurethral routes. Japan was the first country to approve oral 5-ALA administration as a diagnostic agent. The clinical treatment of NMIBC by the Pharmaceuticals and Medical Devices Agency approved ALAGRIO^®,^ which was covered by insurance in 2017. ALAGRIO^®^ was approved to visualize NMIBC during TURBT. The recommended dose of ALA is 20 mg/kg body weight, and ALA-PDD should be initiated approximately 3 h (range: 2–4 h) after oral ALA administration. ALAGRIO^®^ was used in approximately 13,000 cases across 370 institutions in the first 3 years after approval [78].

Transient adverse events for oral ALA include nausea, vomiting, photosensitivity, hypotension, and liver enzyme dysfunction. Our group (Yamamoto et al.) revealed that among the 76 patients included, 7 (9.2%) experienced hypotension (systolic blood pressure (SBP) of <80 mmHg or a mean arterial pressure of <60 mmHg), with a median onset time of 9 (range: 3–28) min after inducing anesthesia [84]. Severe hypotension that sometimes requires management under an intensive care unit after ALAGRIO^®^ oral administration has been reported in Japan [85,86,87]. In a previous study, our group (Fukuhara et al.) reported a combination of factors in the prediction model for ALA-induced hypotension using the detection tree analysis, as follows: (i) general anesthesia, age of ≥74 years, and an American Society of Anesthesiologists physical status of ≥2; (ii) an SBP of ≤115 mmHg at the beginning of anesthesia under spinal anesthesia; and (iii) an SBP of ≥115 mmHg at the beginning of spinal anesthesia and an estimated glomerular filtration rate of ≤42 mL/min/1.73 cm^2^ [88]. Table 1 summarizes the risk factors of 5-ALA-induced hypotensive attack. Despite no treatment, liver dysfunction in response to ALAGRIO^®^ was transient and gradually normalized [88].

Adding PDD and narrow-band imaging (NBI) to WL increases the cancer detection rate for flat lesions, which are difficult to detect with conventional WL [89,90,91,92]. A systematic review by Chen et al. indicated that NBI is a more sensitive diagnostic intervention for patients with NMIBC compared with either 5-ALA or hexylaminolevlinate (5-ALA derivative), based on diagnostic test accuracy assessment using WL cystoscopy as the reference standard [93]. However, Hagimoto et al. revealed that ALA-PDD was more sensitive than NBI for CIS lesions (94.6% vs. 54.1%) [94]. Hagimoto et al. reported a specificity of 22.5% for 5-ALA-PDD in BC [94]. However, according to Fukuhara et al., the specificity of 5-ALA in BC varies among reports, ranging from 48% to 80.6% in a review of six papers [95]. Table 2 summarizes the sensitivity of 5-ALA-PDD in CIS and non-CIS BC in WL, NBI, 5-ALA-PDD, and WL + PDD. NBI improves the cancer detection rate (*p* = 0.033), but its effects on reducing the recurrence rate of BC are unclear, except in patients at a low risk (pTa, grade 1, <30 mm, and no CIS) [96]. The Clinical Practice Guidelines for Bladder Cancer in Japan (2019 revision) recommended PDD because it lowers the recurrence rate of BC more than NBI [97].

## 7. PDD with 5-ALA for TURBT in NMIBCs

ALA-PDD is an effective method for diagnosing NMIBC. Fluorescence light from 5-ALA and WL was previously reported to detect CIS lesions with sensitivities of 92.1% and 47.4%, respectively, and ALA-PDD was significantly more sensitive [84]. However, false-positive lesions increased due to the tangent effect in some distal bladder lesion locations, including the bladder neck, trigone, and prostatic urethra [98]. Thus, ALA-PDD demonstrated a lower specificity compared with WL. However, the higher sensitivity of ALA-PDD compared with WL enables more tumor and CIS lesion detection [97]. In a previous study comprising a colorectal cancer patient with peritoneal dissemination, a small, flat lesion that went undetected under WL was subsequently detected by ALA-PDD [99]. Likewise, in a murine model of peritoneal biliary cancer, micro-dissemination nodules (<1 mm) that remained undetected by WL were detected by ALA-PDD [100]. Thus, compared to WL, ALA-PDD may detect more imperceptibly small tumors and CIS lesions in BC.

**Figure 3:**
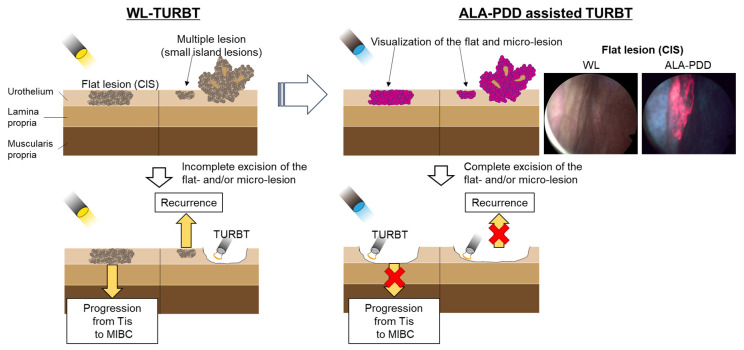
Comparison between WL and 5-ALA for TURBT. PDD-assisted TURBT with 5-ALA increased the detection rate of tumors, particularly for CIS. Additionally, PDD-assisted TURBT with 5-ALA reduced the risk of disease recurrence more than WL-TURBT in patients with NMIBC.

TURBT is the standard surgical treatment for NMIBC. However, conventional TURBT with WL may not detect 4–41% of small papillary tumors, CIS, multifocal growth, and microscopic lesions [101,102]. Several systematic reviews revealed that PDD-assisted TURBT with 5-ALA or its derivative (hexaminolevulinic acid) increased the detection rate of tumors, particularly for CIS. Additionally, PDD-assisted TURBT with 5-ALA reduced the risk of disease recurrence more than WL-TURBT in patients with NMIBC [103]. Figure 3 shows these concepts.

In a systematic review by Veeratterapillay et al., a meta-analysis revealed that the 1-year recurrence-free survival (RFS) after ALA-PDD ranged from 50.4% to 89.6% (vs. 39.0% to 85.0% for WL), and the 2-year RFS ranged from 40.0% to 89.6% (vs. 28.0% to 72.0% for WL) [104]. Furthermore, a meta-analysis of 1782 patients revealed an increased risk of recurrence in patients undergoing WL-guided TURBT at 1 year compared to those receiving PDD (hazard ratio [HR], 1.14; 95% CI, 1.05–1.23; I^2^ = 70%; *p* = 0.002). Moreover, a meta-analysis of 925 patients showed the same tendency after 2 years (HR, 1.25; 95% CI, 1.15–1.35; *p* ≤ 0.001) [104]. These results indicate that the concomitant use of PDD increases the RFS. Matsushita et al. reported a significantly longer RFS in the PDD group in all subgroups except for the tumor size compared with the WL group after a 1:1 propensity score matching of 383 patients for age, sex, concomitant history of upper urinary tract UC, preoperative cytology, tumor multiplicity, and tumor size [105]. Moreover, in a study of 1578 consecutive patients with primary NMIBC, Miyake et al. revealed that PDD-TURBT decreased the risk of high- and low-grade tumor recurrences compared with WL-TURBT. PDD significantly reduced the risk of International Bladder Cancer Group-defined progression in patients with NMIBC [106]. However, PDD-TURBT did not reduce recurrence in patients with NMIBC of ≥30 mm [106,107]. The 5-ALA-induced stability of PpIX is dependent on the wavelength and intensity of the right side. PpIX elimination, called photobleaching, is accelerated during WL cystoscopy [101]. Thus, PDD-TURBT did not reduce recurrences in patients with large tumors that require the long-term use of WL cystoscopy. Yamashita et al. showed that, in gastric tumors, 5-ALA-PDD using a spectrometer with a liquid crystal variable filter and a mean fluorescence excitation with 660–700 nm, in which the photobleaching effect is lower, instead of a peak altitude of 630 nm, was more effective at distinguishing tumors from surrounding nontumoral tissue [108]. Although there are no reports of the application of this technique to BC to our knowledge, this technique may improve the photobleaching effect not only in gastric tumors but also in NMIBC’s ALA-PDD. Intravesical BCG instillation induces bladder wall inflammation. This immune response activation induces and supports antitumor mechanisms [109]. BCG therapy prevents intravesical recurrence and progression, thereby prolonging survival in patients with high-grade NMIBC [110]. However, BCG-induced inflammation may cause false-positive fluorescence in PDD. Draga et al. indicated that >3 months of BCG instillation before fluorescence cystoscopy decreases PDD specificity [111]. However, a study of 99 patients by Nakagawa et al. revealed that the combined use of PDD and BCG improved the identification and resection of tumors, especially those that could not be identified by WL alone and may prolong RFS, although the PDD + BCG (PDD-TURBT followed by BCG treatment) group demonstrated a higher risk and a high CIS detection rate [112]. Nakagawa et al. proposed several factors that may influence RFS: (i) BCG uptake may be more emphasized at the tumor resection site, resulting in a better therapeutic effect; and (ii) The use of PDD may cause a photodynamic therapeutic effect and may contribute to the extension of RFS [112,113,114]. In the BCG combination therapy group, the presence or absence of PDD did not show a significant difference in 2-year progression-free survival, and PDD did not affect disease progression control [112]. Makino et al. revealed that prior BCG infusion history, particularly BCG-unresponsive disease, is responsible for poor prognostic factors in NMIBC patients undergoing PDD-TURBT [115]. Thus, special postoperative follow-up may be needed for these patients.

## 8. PDT with 5-ALA for TURBT in NMIBCs

NMIBC is an excellent target for ALA-PDT. As indicated in the review by Rahman et al., the reasons for this are as follows: (i) PDT may be a good option for the treatment of BC because bladder lesions are readily accessible by endoscopy and bladder tissue exhibits high optical transparency; (ii) Longer-wavelength light has a deeper penetration depth and, at the same time, increases the potential for intrinsic muscle damage, resulting in impaired bladder activity; (iii) PDT, such as irradiating the entire bladder with red light (630 nm) after the intravenous administration of Photofrin, may damage the muscle layer and cause bladder dysfunction; and iv) In contrast, PpIX, which produces 5-ALA or HAL, is excited by green light, which does not penetrate more than 1 mm into the bladder; thus, bladder function may be preserved [116]. The choice of a photosensitizer and excitation light is important. Flat BC demonstrates high recurrence rates. Thus, most patients require additional treatment (e.g., CT and BCG) to prolong recurrence-free intervals and prevent recurrence and disease progression. However, additional intensive care is often necessary after combined modality treatments (such as TURBT + BCG and TURBT + CT) to treat complications associated with chemotherapeutic agent toxicity or BCG-induced severe inflammation [117,118]. The antitumor effects of ALA-PDT, which targets accumulated PpIX in tumor cells, are induced by low-energy stimulation, which is painless to the patient, does not require anesthesia, and may be performed repeatedly [119]. Kriegmair et al. first used 5-ALA in PDT for BC in 1996. Subsequent clinical studies were conducted on patients with treatment-resistant BC and bladder CIS using excited green (514 nm), red (630–635 nm), and WL (380–700 nm) [114,120,121,122,123,124,125]. A study of 45 patients by Filonenko et al. revealed that intraoperative ALA-PDT after TURBT significantly reduced the 1-year recurrence rate for superficial BC (pT stage: Ta-T1) compared with the recurrence rate after TURBT monotherapy (22% vs. 40–80%). These results were at least equivalent to those of convenient adjuvant treatments in patients with bladder tumors (the 1-year recurrence rates were 36–44% after TURBT + CT and 20–59% after TURBT + BCG) [126]. ALA-PDT acts on a neoplastic cell attributively and is highly accurate, minimally invasive, and widely applicable. Further improvements, such as a wavelength or the luminescence sensitizer, may be necessary, but ALA-PDT may replace additional treatments for BC, especially CIS. Moreover, large trials with long-term follow-ups should confirm the beneficial effects of ALA-PDT for BC. Skyrme et al. reported a lower recurrence rate with the combination of mitomycin C and ALA-PDT, suggesting that the sequential administration of mitomycin C and ALA-PDT is safe, well tolerated, and may be effective in the treatment of difficult-to-control superficial transitional epithelial and bladder intraepithelial cancers [125]. Table 3 summarizes the results of the cited literature examining the outcomes of 5-ALA-PDT by tumor stage in NMIBC.

## 9. Screening Using 5-ALA after TURBT in NMIBCs

Urine cytology is frequently performed to screen for BC. Generally, urine cytology is frequently problematic because of its sensitivity in low-grade BC. Interestingly, Yamamichi et al. revealed that 5-ALA-induced fluorescent-selective upper tract urinary cytology, by which the urine sample was centrifuged and the pellets were suspended in minimum essential media using 5-ALA hydrochloride, was more sensitive than conventional cytology for diagnosing upper urinary tract UC (90.4% vs. 66.3%, *p* < 0.001), regardless of the pT and tumor grade [127]. In addition, 5-ALA-induced fluorescent cytology (AFC) for BC showed greater sensitivity than conventional cytology (83.3% vs. 67.9%, *p* = 0.030) [128]. Table 4 summarizes the sensitivity and specificity of conventional cytology and AFC for BC in the literature [128,129]. In addition to AFC, several other urinary biomarkers (e.g., bladder tumor-associated antigen (BTA), the nuclear matrix protein (NMP) 22, and the UroVysion test) are known. Yamamichi et al. reviewed the sensitivity and specificity of these tests and reported the following values: 53–89% and 53–89%, respectively, for BTA, 32–92% and 51–94%, respectively, for NMT22, and 50–71% and 66–72%, respectively, for UroVysion; the corresponding values for 5-ALA were 74–86% and 70–100%, respectively [128]. The test for 5-ALA had the lowest cost and the shortest test time compared to other urinary biomarker tests. Nakai et al. found that the positive rates of low-grade and high-grade lesions by AFC and hexaminolevulinate-induced fluorescent cytology (HFC) were 56% and 96% and 52% and 100%, respectively, compared to 15% and 52% by conventional cytology, using the spectrophotometric photodynamic detection method for BC [130]. In NMIBC, the positivity rates for Ta, CIS, and T1 were 56%, 100%, and 89%, respectively, by AFC, and 52%, 100%, and 100%, respectively, by HFC compared to 15%, 67%, and 33%, respectively, via conventional cytology [130]. Cystoscopy remains necessary as a follow-up procedure, but these results suggest that fluorescence-induced cytology and PDS may be effective for follow-up after TURBT in NMIBC and low-grade lesions.

## 10. Conclusions

NMIBC and MIBC exhibit great intrinsic heterogeneity with regard to the different prognoses, survival, progression, and treatment outcomes. However, it is crucial to decrease tumor recurrence in BC (especially in CIS and pT1 BC) by detecting the range of the lesion and removing it completely.

5-ALA, a new-generation photosensitizer, exhibits a high tumor specificity. In addition, it occurs naturally in the body and is produced from glycine and succinyl CoA. 5-ALA is used in ALA-PDD and ALA-PDT. ALA-PDD has a significantly higher sensitivity in detecting tumors than WL-PDD and NBI, especially in CIS. However, it produces false-positive findings in the trigone and neck portion of the bladder by enhancing red fluorescence in the direction of the tangent, the so-called tangent effect. Fukuhara et al. markedly reduced this false-positive rate due to the tangent effect from 41.2% to 17.4% by modifying the device from a rigid to flexible fluorescent cystoscope; this enabled the perpendicular observation in the bladder neck and trigone without a loss of sensitivity [131]. These systems provide twin-mode PDD (i.e., simultaneous observation of both WL and blue light modes on two screens), through which a physician can observe the red fluorescent image while comparing it to the WL mode image and quickly access the target area; photobleaching in the red-fluorescent regions can be prevented in this manner [131]. We believe that such device refinements would significantly reduce the risk of the recurrence of TURBT with ALA-PDD in NMIBC and decrease progression.

The application of ALA-PDT in the ALA-related field for cancer has potential due to its spatiotemporal selectivity, low toxicity, noninvasiveness, and minimal side effects. However, although ALA-PDD has been successfully applied in many countries, the clinical application of ALA-PDT has not progressed well [132]. As mentioned above, 5-ALA is converted to PpIX via the heme synthesis pathway and accumulates in tumor cells to become a prodrug for PDT. However, 5-ALA is very hydrophilic at the physiological pH, making it difficult to penetrate cells via the biological membranes [133]. Moreover, it is unstable and prone to dimerization under weak alkaline conditions [134], limiting PpIX accumulation and hindering the efficiency and potential applications of 5-ALA-PDT. Therefore, the clinical application of ester derivatives of ALA, such as methyl ester (Me-ALA) and hexyl ester (He-ALA), which increase the lipophilicity of ALA, has been investigated not only in ALA-PDD but also in ALA-PDT [132]. According to a review by Casas et al., in Europe and the United States, Me-ALA is approved for use in dermatology to treat actinic keratosis, basal cell carcinoma, and Bowen’s disease [132]. In BC, fluorescence induced by ALA or He-ALA has been devoted to assisting TURBT and has not been used for radical ALA-PDT [133]. 

In addition to the lipophilization of ALA, several in vitro approaches have been tried to enhance the effect of ALA-PDT. Nakayama et al. attempted to arrest the cell cycle of the human UC cell line at the G2/M phase and accumulate PpIX by co-administering ALA-PDT with mitomycin C [135]. Zhang et al. showed that cell phototoxicity is strongly correlated with intracellular PpIX fluorescence levels, indicating the potential application of the ALA-HPO conjugate in ALA-PDT [136]. However, the clinical efficacy of ALA-PDT for advanced-stage BC is currently limited. ALA-PDT is a superficial, noninvasive procedure; hence, it can prevent muscle damage-induced bladder dysfunction. CIS is a precursor lesion to invasive cancer, a superficial lesion, and sometimes difficult to detect. Moreover, as mentioned above, 95% of superficial bladder tumors do not respond to BCG progress in 5 years [5]. In addition, the standard of care in patients with BCG-nonresponsive NMIBC is radical cystectomy [137]. Thus, NMIBC, especially CIS, can be an excellent imminent target for ALA-PDT. Cancer cells, including BC, produce ATP through the anaerobic pathway of glycolysis rather than the oxidative phosphorylation pathway in the TCA circuit and the electron transfer system. This phenomenon is called the Warburg effect. Griglavicius et al. showed that 5-ALA inhibited lactate dehydrogenase (LDH) in the glycolytic system; additionally, 5-ALA-PDT improved the therapeutic efficacy of PDT by approximately 15% in Warburg-type glioblastoma multiforme cell lines, with and without prior LDH inhibition with tartonate (a known LDH inhibitor) [138]. This finding is based on the Warburg effect, which is a fundamental property across all cancers and is expected to have a similar effect in BC, where the specific accumulation of 5-ALA is much higher than that in surrounding normal tissues. With these technological advances, 5-ALA-PDT is expected to preserve bladder function and have a potent disabling effect limited to tumor cells. It may become an extremely powerful procedure in the treatment of MNIBC, particularly CIS.

Although the ALA-related field is useful for NMIBC, hypotension is well known as a transient adverse event for oral ALA. Our group revealed the multiple factors that are mutually related in the prediction model for ALA-induced hypotension, as mentioned above, and showed the possibility of the conquest of this side effect. Particular attention should be given to such patients. One of the possibilities for preventing hypotension after the induction of anesthesia is to discontinue renin-angiotensin system inhibitors in patients taking them [86]. The success of clinical ALA-PDT for NMIBC and its response to side effects, as with ALA-PDD, may be developed by clinical experience and a deeper understanding of the pathobiology of NMIBC. 

**Figure 4:**
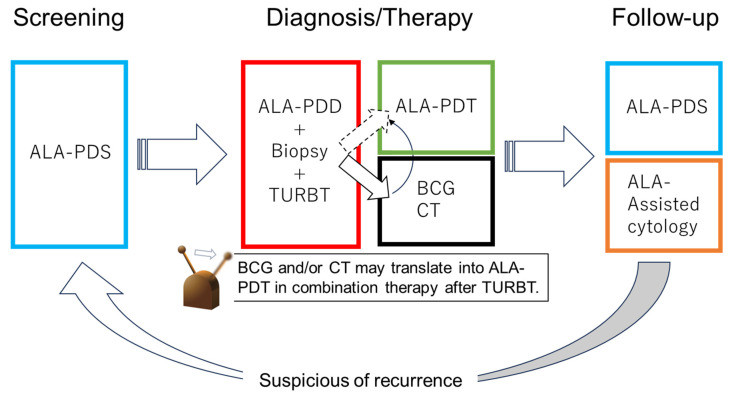
Future perspective of 5-ALA in NMIB. In the future, bladder cancer diagnosis, treatment, and follow-up may be performed with 5-ALA.

With future technological advances, ALA-PDT may replace additional BC treatments, such as CT and BCG. Patients with NMIBC can be treated with PDD-TURBT and ALA-PDT and then receive follow-up using ALA-PDS and ALA-induced fluorescent urinary cytology. Thus, the diagnosis, treatment, and follow-up of BC may be performed using 5-ALA in the future (Figure 4).

## Figures and Tables

**Table 1:** A summary of the risk factor of 5-ALA-induced hypotension.

Authors, Year [Reference]	Risk Factors of 5-ALA-Induced Hypotensive Attack
Nohara et al., 2019 [86]	▪ General anesthesia (OR 6.240) ▪ Regular renin-angiotensin system inhibitor use (OR3.378)
Yatabe et al., 2020 [87]	▪ Female sex (OR 3.95) ▪ Systolic blood pressure < 100 mmHg before anesthesia induction (OR 13.30) ▪ General anesthesia (OR 25.84)
Fukuhara et al., 2021 [88]	▪ History of hypertension (OR 7.568) ▪ General anesthesia (OR 14.435) [the detection tree analysis] i General anesthesia + age of ≥74 years + ASA-PS ≥ 2 ii SBP of ≤115 mmHg at the beginning of anesthesia under spinal anesthesia iii SBP of ≥115 mmHg at the beginning of spinal anesthesia + an estimated glomerular filtration rate of ≤42 mL/min/1.73 cm^2^

OR: odds ratio; ASA-PS: American Society of Anesthesiologists physical status; SBP: systolic blood pressure; e-GFR: an estimated glomerular filtration rate.

**Table 2:** Sensitivity of WL, NBI, and 5-ALA-PDD in CIS and non-CIS lesions.

Authors, Year [Reference]	Cancer Stage	Number of Lesions	Sensitivity (%)				Stage, Grade Condition
			WL	PDD	NBI	WL + PDD	
Yamamoto et.al., 2020 [84]	CIS	35	47.4	92.1	NR	92.1	NR
	Non-CIS	124	71.0	89.5	NR	93.5	
Hagimoto et al., 2021 [94]	All BC	202	88.1	89.6	76.2	99.5	Ta: 125, T1: 26 T2: 14, CIS: 37 Low grade: 16 High grade: 186
	CIS	37	64.9	94.6	54.1	100	
	Non-CIS	165	92.7	87.9	81.2	98.2	

WL: white light; PDD: photodynamic diagnosis; NBI: narrow-band imaging; NR: not reported.

**Table 3:** Outcomes in NMIBC patients following 5-ALA-PDT.

Authors, Year [Reference]	Patients *n*	Stage before PDT	Grade before PDT	Further Treatment *n*	Response to PDT *n* (mo: Month of Recurrence)	Follow-Up (Month) (Mean)
Kriegmair et al., 1996 [120]	6	Ta	G1-2	TURBT: 2 2nd PDT: 1	CR: 2 PR:1 NC: 3	6–30 (16.5)
	4	CIS + Ta	G2-3	TURBT: 1 TURBT + 2ndPDT: 1 Nd: YAG: 1	CR: 2 PR: 1 PD: 1	9–27 (15.8)
Waidelich et al., 2003 [121]	2	Ta	G1:1 G2:1	TURBT: 1	CR: 2	22–23 (22.5)
	7	CIS	NR	Further treatment: 0	CR: 4 not CR: 3	3–25 (15.3)
	2	Ta + CIS	G1:1 G2:1 G3:1	TURBT: 1	CR: 1 not CR: 1	6–18 (12.0)
Berger et al., 2003 [123]	6	Ta	NR	NR	CR: 3 not CR: 3	43.5
	6	Ta + CIS	NR	NR	CR: 3 not CR: 3	18.8
	1	CIS	NR	NR	CR: 1	26.0
	15	T1	NR	NR	CR: 7 not CR: 8	15.6
	3	T1 + CIS	NR	NR	CR: 2 not CR: 1	33.7
Skyrme et al., 2005 [125]	4	Ta	G2:2 G3:2	administration of mitomycin C	Recurrence: 3 G2: 1 (18 mo) G3: 2 (8, 24 mo)	24
	2	CIS	NR	administration of mitomycin C	Recurrence: 1 (24 mo)	24
	15	T1	G1:8 G2:7	administration of mitomycin C	CR: 3 (G1), 4 (G2) Recurrence: 4 G1: 2 (12, 18 mo) G2: 2 (12 mo)	24

Patients had a history of the transurethral resection of bladder tumors (References [120,121,123]) or the administration of mitomycin C ([125]). Nd: YAG: Neodymium YAG laser; CR: Complete remission; PR: Partial remission; NC: No change; PD: Progressive disease; NR: Not reported; MO: Month.

**Table 4:** Sensitivity and specificity of conventional cytology and 5-ALA-induced fluorescent cytology in BC.

Authors, Year [Reference]	Sensitivity		Specificity	
	Conventional % (*n*)	5-ALA % (*n*)	Conventional % (*n*)	5-ALA % (*n*)
Yamamichi et al., 2019 [128]	67.9 (57/84)	83.3 (70/84)	95.6 (151/158)	96.2 (152/158)
Shadab et al., 2021 [129]	64.0 (16/25)	100.0 (25/25)	96.0 (72/75)	98.67 (74/75)
